# Three‐Dimensional Architecture of Ectopic Epithelium and Vasculature in Ovarian Endometriosis Revealed by Tissue‐Clearing Imaging

**DOI:** 10.1111/cpr.70197

**Published:** 2026-03-12

**Authors:** Fanyuan Sun, Shuzhen Wang, Jinyan Zhao, Jinhua Leng, Xili Zhu, Chongdong Liu, Jing Chen, Jinming Guo, Bangjing Jiang, Haihe Zhou, Zhuoying Li, Hua Li, Menghui Li, Yongcun Qu

**Affiliations:** ^1^ Capital University of Physical Education and Sports Beijing China; ^2^ Department of Obstetrics and Gynecology Beijing ChaoYang Hospital, Capital University of Medical Science Beijing China; ^3^ Department of Obstetrics and Gynecology, Peking Union Medical College Hospital Chinese Academy of Medical Science Beijing China; ^4^ Institute of Zoology, Chinese Academy of Sciences Beijing China

## Abstract

Ovarian endometriosis (OEM) is characterised by ectopic endometrial tissue growth within the ovary. In these ectopic lesions, the ectopic epithelium plays a crucial role in OEM progression and has been associated with malignant transformation in a subset of cases. However, conventional histology limits understanding of ectopic epithelial distribution, structure, and its perivascular microenvironment, thus impeding pathogenesis studies. To address this, we employed a modified tissue‐clearing method and  three‐dimensional (3D) imaging to systematically characterise OEM, revealing key, previously unreported spatial characteristics. We found significantly higher densities of ectopic epithelium and vasculature in the outer cystic wall versus the inner. Furthermore, our method improved the detection rate of ectopic epithelium and revealed its morphological polymorphism at both tissue and cellular levels. Besides, we demonstrated that vessels preferentially cluster around ectopic epithelium, with their distribution pattern strongly linked to the location of ectopic epithelium. Strikingly, we observed endometrial‐like structures in lesional vasculature in 3 of 49 cases, representing a novel morphological observation that warrants further investigation. This study significantly advances our understanding of OEM histopathology, offering insights for clinical diagnosis and treatment.

## Graphical abstract

Our study employed a modified tissue‐clearing method combined with deep imaging for 3D reconstruction of ovarian endometriosis (OEM) lesions, revealing its morphological polymorphism at tissue and cellular levels. Besides, vessels preferentially clustered around the ectopic epithelium, with their distribution pattern strongly linked to the location of the ectopic epithelium.

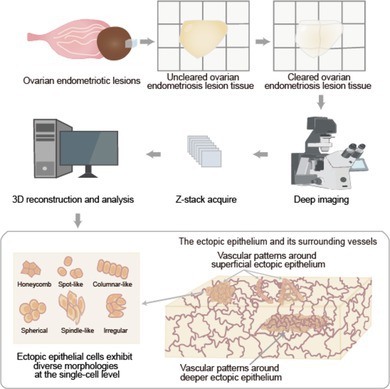

## 1 Introduction

Ovarian endometriosis (OEM) is characterised by the presence of ectopic endometrial tissue in the ovary, severely impacts women's health, causing chronic pain and infertility [1, 2, 3, 4, 5, 6, 7, 8]. The ectopic epithelium within these lesions is central to pathogenesis, as a subset of its cells drives persistent inflammation and harbours cancer‐associated genomic alterations reported in subsets of these lesions [9, 10, 11, 12, 13, 14]. Its function is likely sustained by a specialised vascular niche. This dependence is supported by our prior observation in murine endometrium, where physiological epithelium is closely enveloped by a dense vascular network [15]. We therefore hypothesise that a similarly supportive vascular microenvironment may be established. However, traditional two‐dimensional (2D) histology fails to capture the three‐dimensional (3D) architecture of this epithelium and its spatial relationship with vasculature, limiting pathophysiological insight and therapeutic development.

Tissue‐clearing technology, which enables high‐resolution 3D imaging of intact tissues, has evolved from applications in model organisms to human pathology, offering transformative insights in fields such as neuroscience and oncology [16, 17, 18, 19, 20, 21]. However, its use in gynaecological tissues remains limited, partly because clearing efficacy is highly tissue‐specific. Although Yamaguchi et al. successfully applied the CUBIC (Clear, Unobstructed Brain/Body Imaging Cocktails and Computational analysis) protocol to human adenomyosis, revealing an intricate ‘ant colony‐like’ network of ectopic glands, the 3D morphology of OEM lesions has not been systematically explored [22]. Moreover, when we applied the established CUBIC protocol to human OEM tissues, we encountered a technical obstacle: the refractive index‐matched clearing solution (CUBIC‐R) tended to form crystals on the sample surface, compromising imaging quality and stability (Figure S1). This highlighted the need for protocol optimisation tailored to OEM tissues.

To address this, we implemented a modified CUBIC protocol to achieve optical clearing of human ovarian endometriotic lesions, enabling high‐resolution 3D reconstruction of their pathological architecture. We systematically characterised both the distribution and morphological diversity of ectopic epithelium, as well as deciphered its spatial co‐localisation patterns with the vasculature. Unexpectedly, we identified ectopic epithelium‐like structures within blood vessels of ovarian endometriotic lesions, a finding not previously reported in human ovarian endometriosis, representing a novel morphological observation relevant to disease pathophysiology. Collectively, this work significantly advances our understanding of OEM histopathology and pathophysiology.

## 2 Results

### 2.1 The Modified CUBIC Protocol Enables Tissue Clearing and 3D Imaging of Ovarian Endometriosis Lesion Tissue

**FIGURE 1:**
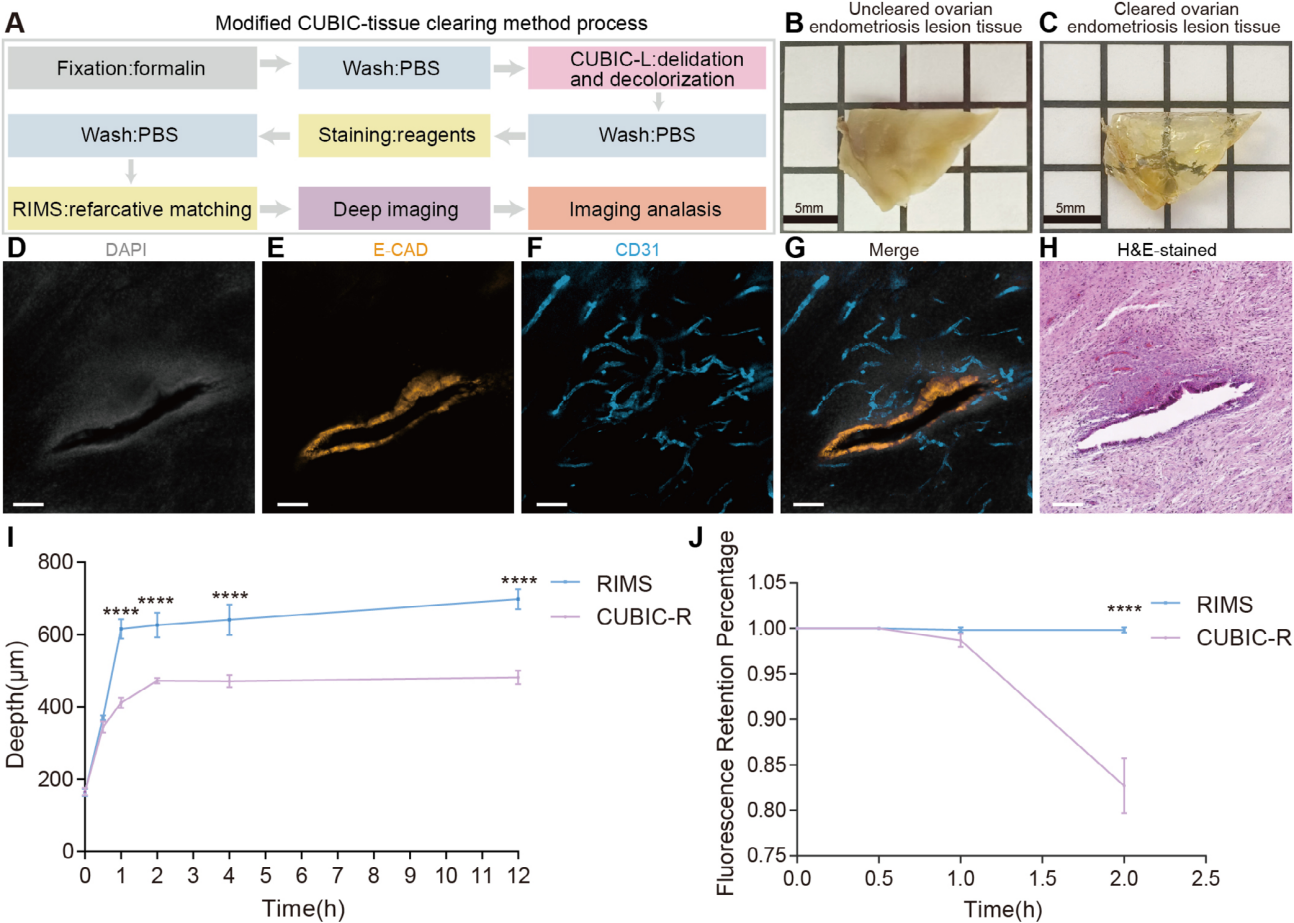
Tissue clearing and imaging of human ovarian endometriosis lesion tissue using modified CUBIC. (A) Schematic diagram depicting the clearing, immunostaining, and imaging process for ovarian endometriosis lesion tissue. (B) Uncleared ovarian endometriosis lesion tissue. Scale bar, 5 mm. (C) Cleared ovarian endometriosis lesion tissue. Scale bar, 5 mm. (D) Image of ovarian endometriosis lesion tissue stained with DAPI (grey) with clearing by modified CUBIC. (E) Image of ovarian endometriosis lesion tissue stained with E‐Cadherin (yellow) with clearing by modified CUBIC. (F) Image of ovarian endometriosis lesion tissue stained with CD31 (blue) with clearing by modified CUBIC. (G) Merged three‐channel visualisation. (H) H&E‐stained image of human ovarian endometriosis lesion tissue. Scale bars: 150 μm (D–H). (I) Light penetration depth of tissue treated with two clearing agents at different time points. (J) Fluorescence retention percentage of tissue cleared by two different agents at different time points during prolonged imaging. Both (I) and (J) were tested at a wavelength of 405 nm.

We collected 49 OEM samples from 49 patients undergoing cyst dissection. In order to achieve tissue clearing in human ovary endometriosis, we implemented CUBIC protocol IV [23], which has demonstrated efficacy in enabling tissue clearing and 3D imaging of adenomyosis [22]. However, the refractive index‐matched clearing solution (CUBIC‐R) readily formed crystals on the tissue surface (Figure S1). This crystallisation not only limited the imaging depth but also led to a rapid decline in fluorescence preservation over time, with signal intensity dropping by 17.3% within 2 h (Figure 1I,J).

To overcome these limitations, we replaced the CUBIC‐R clearing solution with RIMS, due to its superior solubility and established effectiveness in tissue clearing [24, 25]. Utilising this modified CUBIC protocol, we successfully achieved clearing of all human OEM lesions (Figure 1A–C). Compared with the original CUBIC protocol, our modified method achieved a 44.9% improvement in imaging depth whilst maintaining stable fluorescence, enabling high‐quality 3D reconstruction of the entire lesion architecture (Figure 1I). To visualise the structural organisation of the lesions, we performed combined immunostaining using E‐cadherin, a classical and stable marker for delineating epithelial cells in endometriotic lesions [26, 27, 28], and CD31, along with DAPI, to label ectopic epithelial cells, vascular endothelial cells, and nuclei, respectively. Pseudo‐grey colouring allowed us to delineate the intact lesional architecture, whilst pseudo‐yellow and pseudo‐blue colouring enabled visualisation of the spatial distribution and characteristics of ectopic epithelium and vasculature within the lesions (Figure 1D–G). Following tissue clearing and immunostaining, deep optical sectioning of the samples was performed using light‐sheet fluorescence microscopy and confocal microscopy. Compared with the H&E staining (Figure 1H), the optical section in the XY plane showed that modified CUBIC protocol preserved the characteristic 2D morphology of the lesions (Figures 1G and S2A_1_–D_2_). Thus, this approach, combined with subsequent 3D image reconstruction and quantitative analysis using Imaris software (Bitplane), enabled us to map the spatial characteristics of OEM lesions.

### 2.2 Abundant Ectopic Epithelium and Vasculature Are Preferentially Localised to the Outer Cyst Wall

**FIGURE 2:**
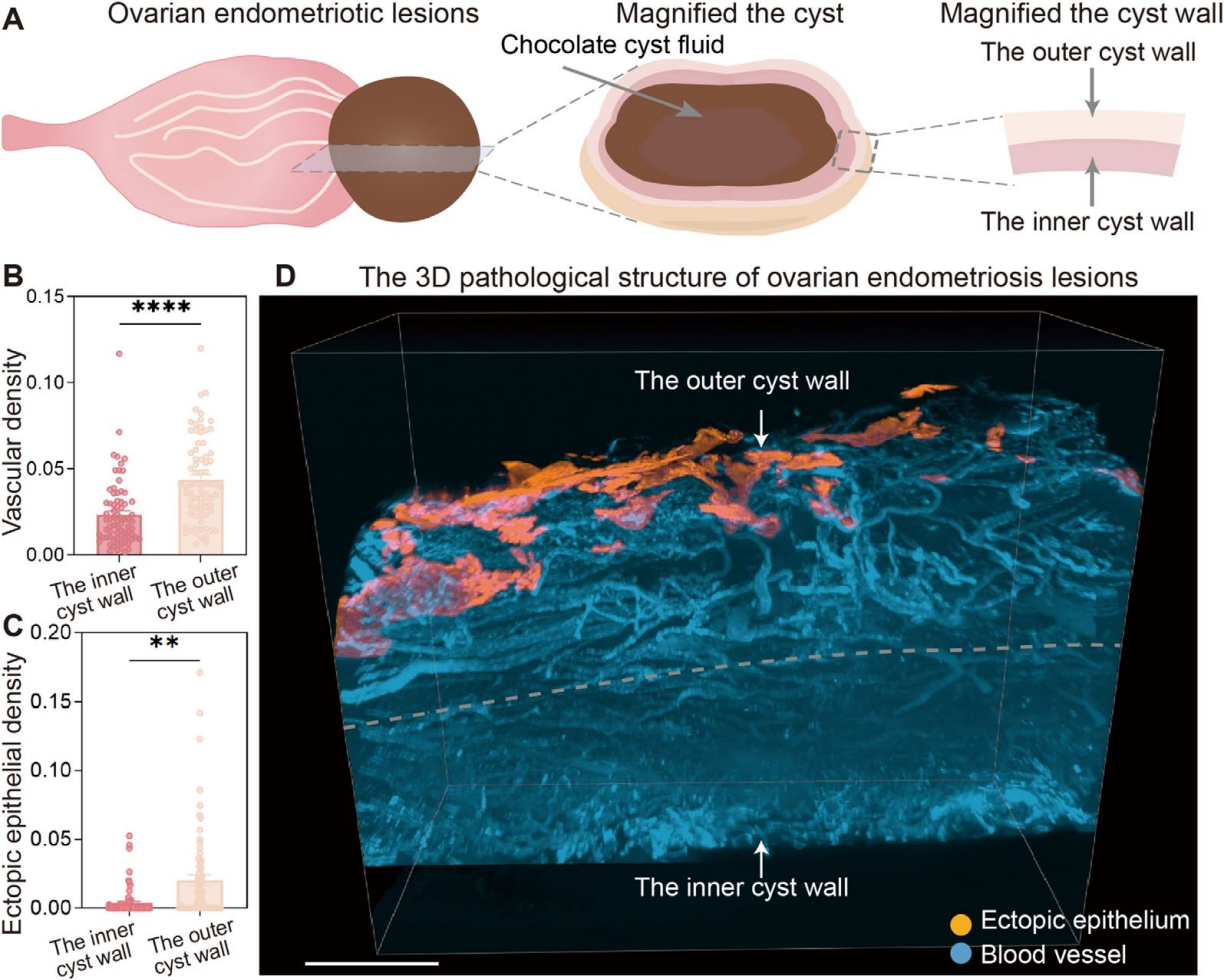
Heterogeneous distribution of ectopic epithelium and blood vessels between inner and outer OEM cyst walls. (A) Schematic diagram of the two‐layer structure of ovarian endometriosis cyst wall observed macroscopically. (B) Comparison of vascular density between the outer and inner cyst walls (*n* = 27). The data were obtained from at least two randomly selected fields of view on the inner and outer cyst walls of each sample. (C) Comparison of ectopic epithelial density between the inner and outer cyst walls (*n* = 27). The data were obtained from at least two randomly selected fields of view on the inner and outer cyst walls of each sample. (D) 3D imaging of blood vessels and ectopic epithelium in ovarian endometriosis lesions. Scale bar: 2.5 mm. Data are presented as mean ± SEM; ****p < 0.0001. **p < 0.05. Statistical analysis was performed using the Mann–Whitney *U* test. Ectopic epithelium (yellow), blood vessel (blue).

Anatomically, ovarian endometriotic cyst walls typically exhibit a distinct two‐layered structure: a brownish inner layer and a whitish outer layer (Figures 2A and S3). To understand the distribution of ectopic epithelium and blood vessels within these two layers, we used light‐sheet microscopy to achieve complete imaging of the inner and outer lesion layers. Following 3D image reconstruction, we observed a significantly greater abundance of ectopic epithelium and blood vessels on the outer wall of the cystic cavity compared to the inner wall (Figure 2D). Quantitative analysis revealed that the density of both ectopic epithelium and blood vessels was significantly higher on the outer wall, with densities 81% and 46% higher, respectively, than that on the inner wall. (Figure 2B,C). These findings demonstrate significant heterogeneity in the distribution of ectopic epithelium and blood vessels between the inner and outer cyst walls in ovarian endometriotic lesions, with predominant localisation to the outer wall.

### 2.3 Ectopic Epithelium Exhibits High Detection Rates and Diverse 3D Organisational Morphologies

**FIGURE 3:**
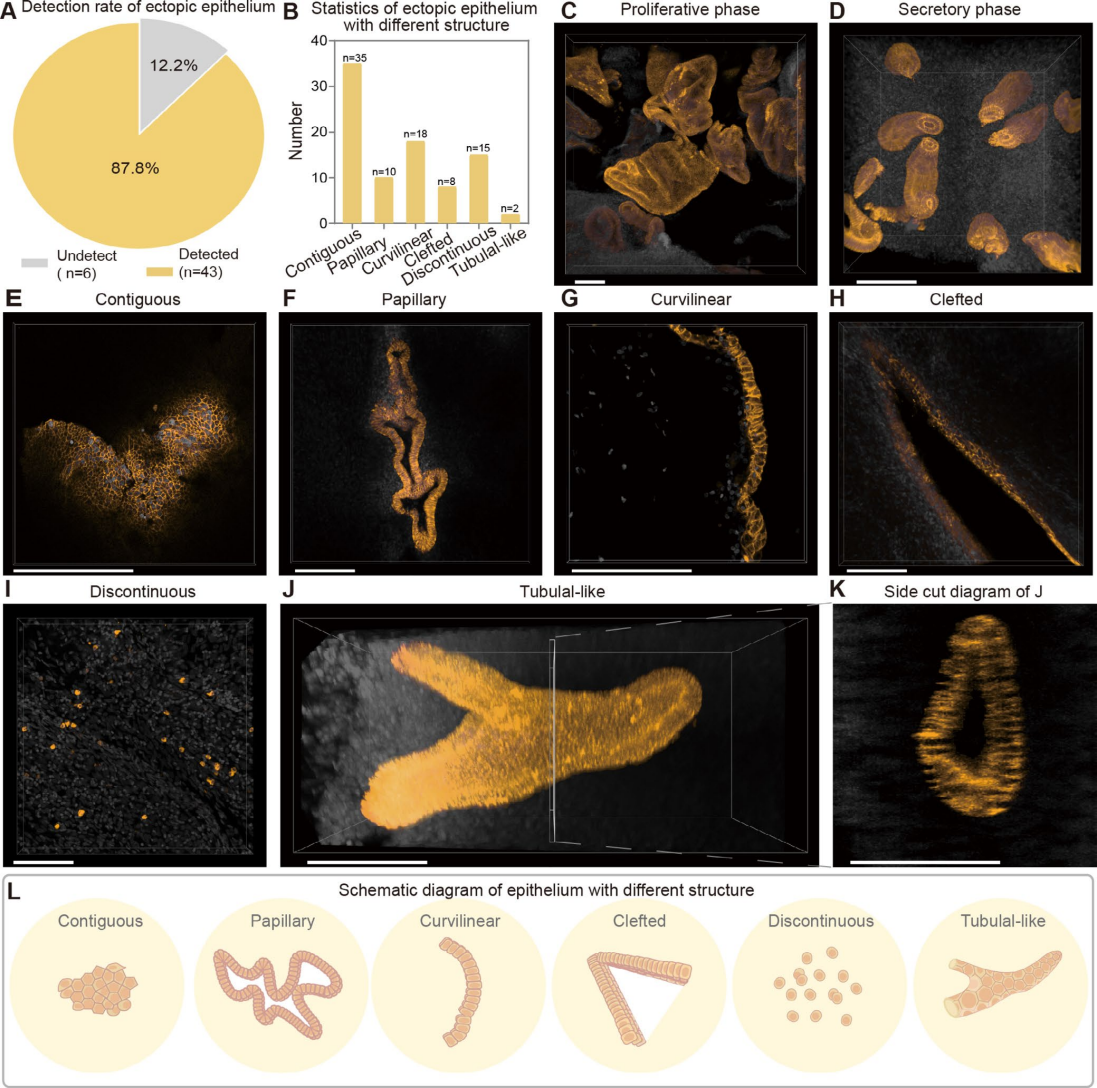
Ectopic epithelium exhibits diverse morphologies at the structural level. (A) Detection rate of ectopic epithelium. (B) Statistical analysis of the number of ectopic epithelium with different morphologies at the 3D level. (C, D) 3D morphology of eutopic epithelium during proliferative (C) and secretory (D) phases. (E–J) Respectively contiguous, papillary, curvilinear, clefted, discontinuous, and tubular‐like ectopic endometrial epithelium. (K) Side cut diagram of (J). (L) Schematic diagram of ectopic epithelium with different structure. Scale bars: 100 μm (C–K). Ectopic epithelium (yellow), nuclei (grey).

Subsequently, we investigated the detection frequency and 3D architectural features of ectopic epithelium in ovarian endometriotic lesions. In our analysis of 49 samples, ectopic epithelium was detected in 43 cases(Figure 3A) (87.8%), a significantly higher detection rate than the 60% previously reported using traditional tissue sectioning techniques [29, 30]. This finding reveals the ubiquitous presence of ectopic epithelium in ovarian endometrioma tissue.

Building on the increased detection rate, our 3D analysis further unveiled a striking heterogeneity in ectopic epithelial organisational morphology. Whilst eutopic endometrial epithelium typically presents a tubular structure [31] (Figure 3C,D), ectopic epithelial tissue exhibited a variety of distinct 3D morphologies, often within a single lesion. As shown in Figures 3E–L and S4A–F, these included: contiguous epithelial structures, characterised by continuous, uninterrupted epithelial layers (Figure 3E); papillary epithelial structures, featuring columnar epithelium with multiple papillary projections (Figure 3F); curvilinear epithelial structures, consisting of single‐layered columnar epithelium arranged in curvilinear configurations (Figure 3G); clefted epithelial configurations, formed by ectopic epithelium on both sides creating an acute, V‐shaped cleft with no intervening stroma (Figure 3H); discontinuous epithelial structures, presenting as discontinuous distributions (Figure 3I); and tubular‐like structures, featuring hollow centres forming luminal structures (some with branching) (Figure 3J,K, Video S1). Statistical analysis of the occurrence frequency of these different structural epithelial clusters revealed that contiguous forms were the most prevalent, whilst tubular‐like forms were the least frequent (Figure 3B). This observation contrasts with the dominance of luminal structures typically observed in normal uterine tissue and indicates a substantial structural remodelling in ectopic epithelium.

### 2.4 Ectopic Epithelial Cells Exhibit Diverse Morphologies and Proliferative Heterogeneity at the Single‐Cell Level

**FIGURE 4:**
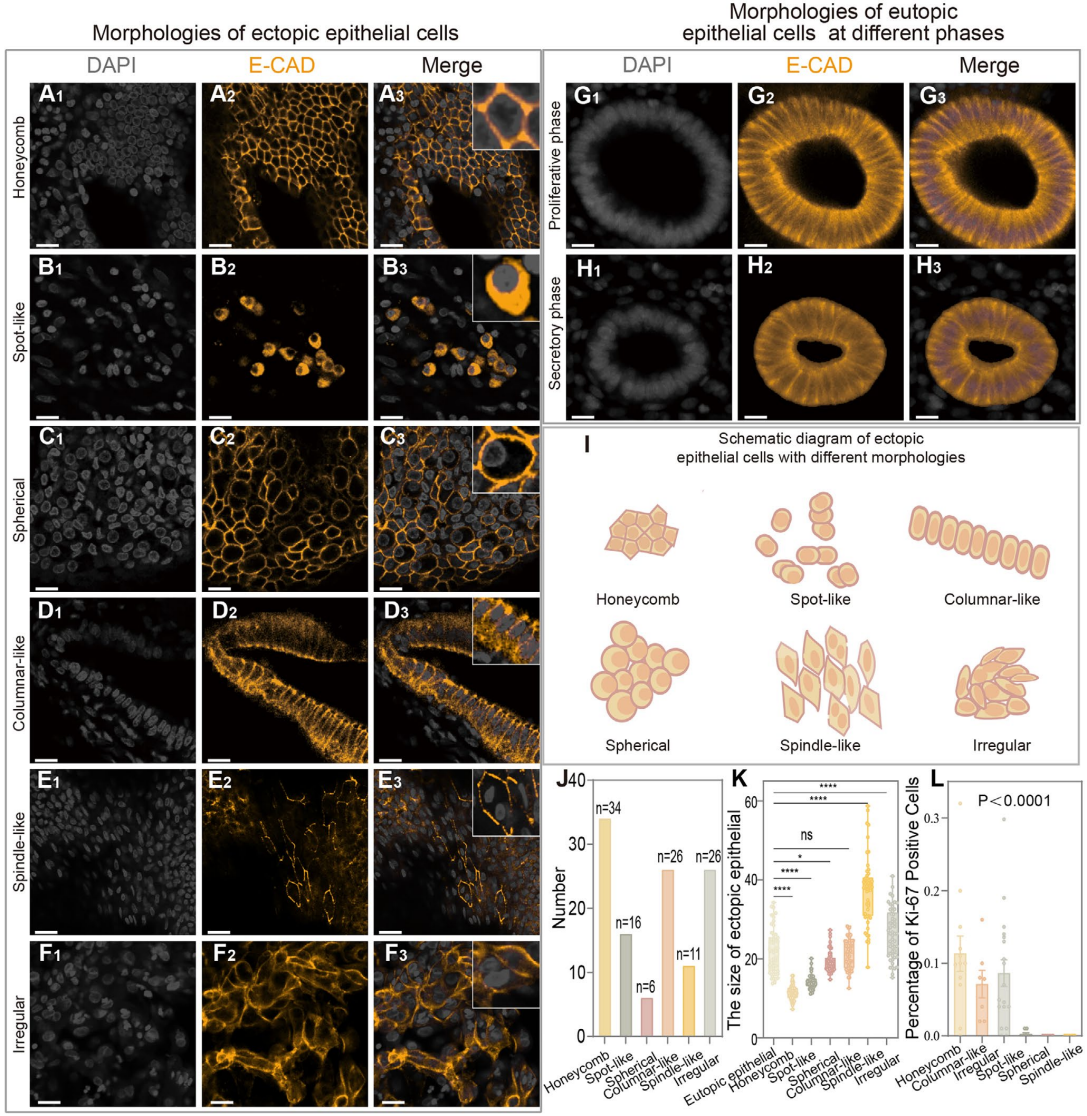
Ectopic epithelial cells exhibit diverse morphologies at the single‐cell level. (A1–A3) Honeycomb ectopic epithelial cells. (B1–B3) Spot‐like ectopic epithelial cells. (C1–C3) Spherical ectopic epithelial cells. (D1–D3) Columnar‐like ectopic epithelial cells. (E1–E3) Spindle‐like ectopic epithelial cells. (F1–F3) Irregular ectopic epithelial cells. (G1–G3) Eutopic endometrial epithelial cells' morphology at proliferative phase. (H1–H3) Eutopic endometrial epithelial cell morphology at secretory phase. (I) Schematic diagram of ectopic epithelial cells with different morphologies. (J) Statistical analysis of the number of ectopic epithelial cells with different morphologies at the single cell level. (K) Quantitative comparison of cell size of epithelial cells (*n* = 2. Measure 25 cells of eutopic epithelial cells in the proliferative and secretory phases, respectively; *n* ≥ 4. Measurement of 50 cells for each morphology of ectopic epithelial cells.) (L) Ki‐67 positive rate of ectopic epithelial cells in different morphological types. Scale bars: 30 μm (A1–H3). Data are mean ± SEM; ****p < 0.0001. Statistical analysis was performed using the Kruskal‐Wallis *H* test. Epithelium (yellow), nuclei (grey).

At the single‐cell level, 3D reconstructions revealed that ectopic epithelial cells in ovarian endometrioma lesions differed fundamentally from eutopic endometrial epithelium. In contrast to the homogeneous columnar morphology consistently observed in eutopic epithelial cells (Figure 4G1–H3), ectopic epithelial cells lacked uniform cellular architecture and instead exhibited pronounced variability in cell shape and spatial organisation. Rather than forming a homogeneous epithelial layer, ectopic epithelial cells preferentially organised into distinct cellular arrangements, a difference supported by quantitative single‐cell measurements, and diverse ectopic epithelial cells preferentially organised into specific ectopic epithelial tissue arrangements (Figure 4K, Table S1). This comparison underscores a fundamental architectural divergence between eutopic and ectopic epithelium.

Based on cell shape, individual ectopic epithelial cells could be classified into multiple morphotypes, including honeycomb, spot‐like, spherical, columnar‐like, spindle‐like, and irregular forms (Figure 4A1–F3). These distinct morphotypes frequently coexisted within the same lesion, indicating substantial cellular‐level heterogeneity within ectopic epithelium. Quantitative single‐cell analysis further revealed distinct structural features amongst these epithelial morphotypes (Figure 4K). Honeycomb cells were relatively small, polygonal, and tightly packed, whereas spot‐like cells were similar in size but spherical, with nuclei occupying a large proportion of the cellular volume (Figure 4A1–B3). Spherical cells were significantly larger than spot‐like cells and displayed eccentrically positioned nuclei (Figure 4C1–C3). Columnar‐like cells exhibited an elongated and ordered morphology reminiscent of eutopic endometrial epithelium (Figure 4D1–D3). In contrast, spindle‐like cells represented the largest morphotype and were characterised by reduced E‐cadherin expression and occasional multinucleation, whilst irregular cells showed variable shapes with enlarged cell and nuclear sizes, also accompanied by sporadic multinucleation (Figure 4E1–F3). Consistent with these observations, honeycomb cells constituted the most prevalent ectopic epithelial population at the single‐cell level (Figure 4J).

To determine whether this morphological heterogeneity was associated with differences in cellular activity, proliferative status was assessed using Ki67 immunostaining. Quantitative analysis revealed significant variability in Ki67 positivity amongst epithelial morphotypes (Figure 4L). Honeycomb, columnar‐like, and irregular cells exhibited the highest proportions of Ki67‐positive nuclei, whereas spot‐like, spherical, and spindle‐like morphotypes consistently showed low proliferative indices. Notably, the three most frequently observed morphotypes (honeycomb, columnar‐like, and irregular) also exhibited amongst the highest Ki67‐positive fractions, suggesting that proliferative heterogeneity within ectopic epithelium is disproportionately associated with dominant cellular configurations rather than uniformly distributed across all epithelial cells. These results indicate that cellular morphotypes identified within ectopic epithelium are associated with distinct proliferative states, supporting the presence of functional heterogeneity at the cellular level.

### 2.5 Vessels Preferentially Surround Ectopic Epithelium in Location‐Specific Patterns

**FIGURE 5:**
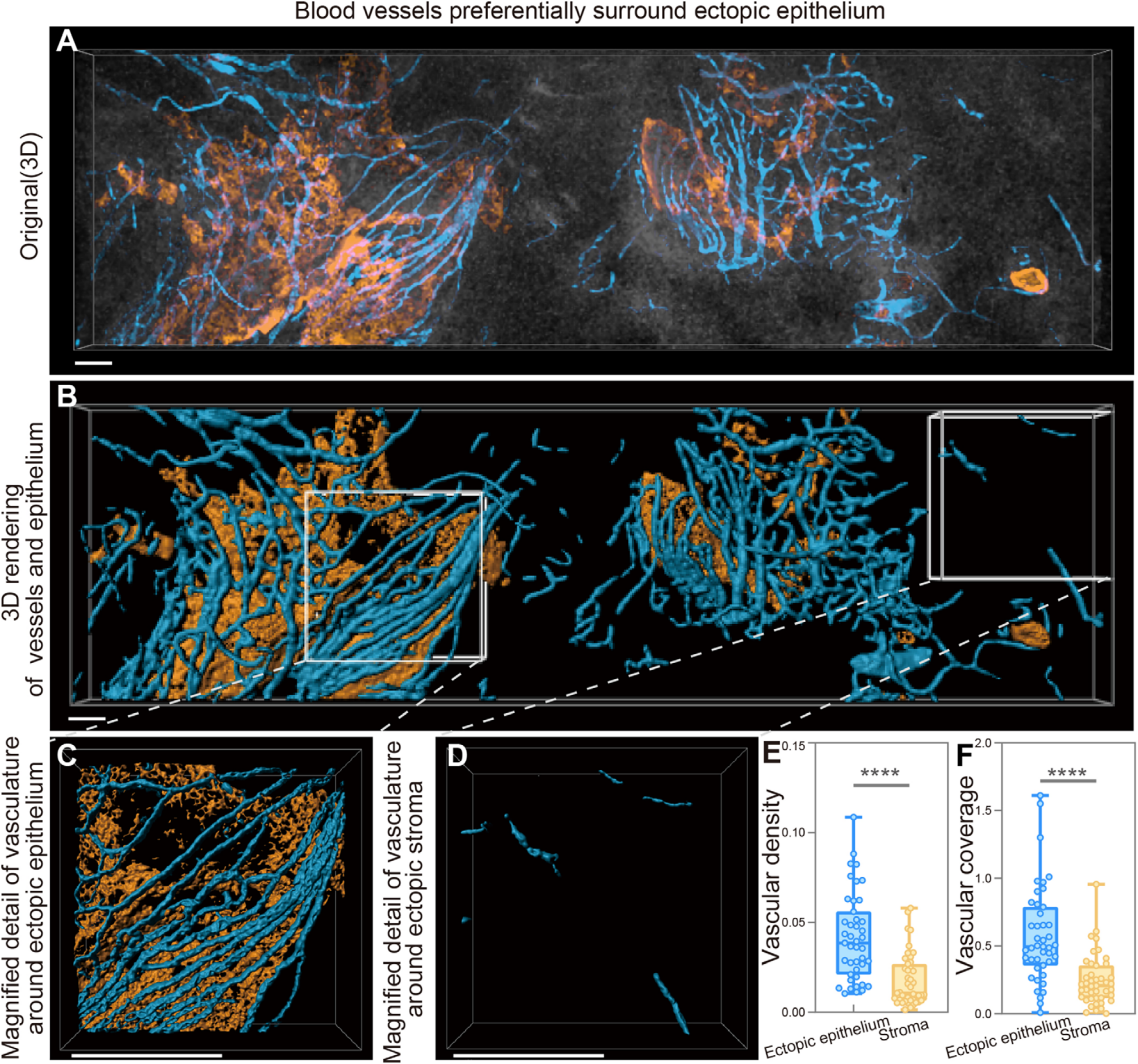
Blood vessels preferentially surround ectopic epithelium in OEM lesions. (A) 3D original image of ectopic epithelium, nuclei, and blood vessels. (B) 3D rendering of vessels and epithelium of (A). (C) Magnified detail of vasculature around ectopic epithelium. (D) Magnified detail of vasculature around ectopic stroma. (E) Comparison of vascular density around ectopic epithelium and stromal tissue. (F) Comparison of vascular coverage around ectopic epithelium and stromal tissue. (*n* = 43). Measurement of the vascular density and vascular coverage surrounding the ectopic epithelium and the vascular density surrounding the stroma. Scale bars: 100 μm (A–D). Data are mean ± SEM; ***p < 0.001. Statistical analysis was performed using the Mann–Whitney *U* test. Ectopic epithelium (yellow), blood vessel (blue), nuclei (grey).

**FIGURE 6:**
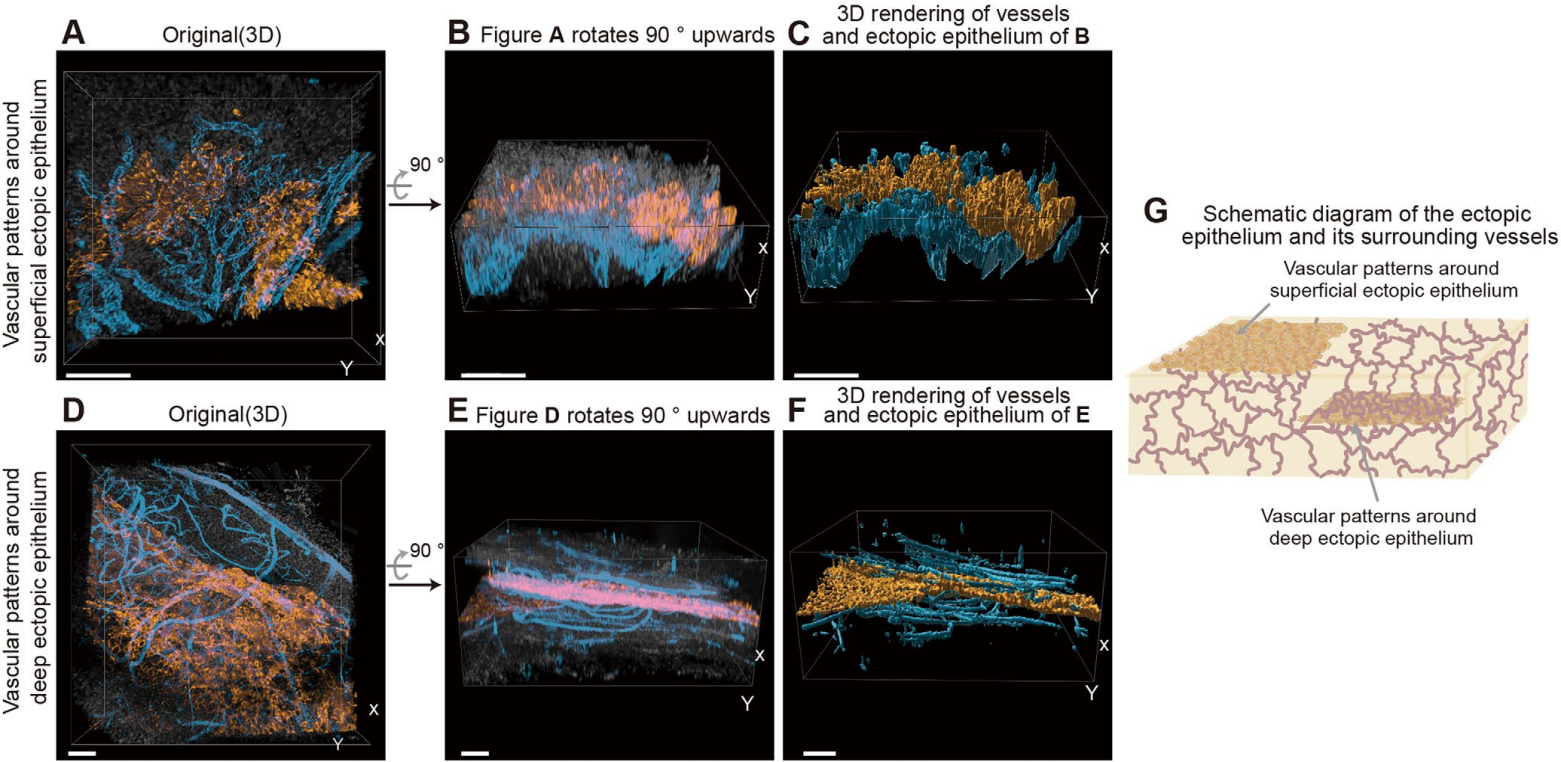
Vascular network distribution patterns are dependent on ectopic epithelium location in OEM. (A) 3D original image of vascular patterns around superficial ectopic epithelium in OEM lesions. (B) 90° rotated view of A. (C) 3D rendering of vessels and ectopic epithelium of B. (D) 3D original image of vascular patterns around deep ectopic epithelium in OEM lesions. (E) 90° rotated view of D. (F) 3D rendering of vessels and ectopic epithelium of E. (G) Schematic diagram depicting the relationship between ectopic epithelium location and vascular patterns. Scale bars: 100 μm (A–F). Ectopic epithelium (yellow), blood vessel (blue), nuclei (grey).

We next investigated the vascular patterns surrounding ectopic epithelium. 3D imaging results showed that blood vessels were preferentially localised around ectopic epithelium (Figure 5A–D, Video S2). Consistent with this observation, statistical analyses revealed that vascular density and vascular coverage around ectopic epithelium were significantly higher than those around the stroma, by 134% and 136%, respectively (Figure 5E,F). Moreover, we observed that vascular distribution is highly dependent on the location of the ectopic epithelium (Figure 6). Specifically, when the ectopic epithelium grows on the surface of the lesion, the surrounding vessels are primarily distributed beneath the ectopic epithelium, forming a unilateral vascular network (Figure 6A–C,G, Video S3). In contrast, when the ectopic epithelium grows in the deeper layers of the lesion, the surrounding vessels mainly encircle the ectopic epithelium, forming a circumferential structure (Figure 6D–G, Video S4). These results suggest a preferential distribution of blood vessels around the epithelium, with their distribution patterns being closely linked to the epithelial location. This observation indicates that epithelial growth and its spatial positioning may influence vascular distribution, thus suggesting a potential cross‐talk between the epithelium and blood vessels.

**FIGURE 7:**
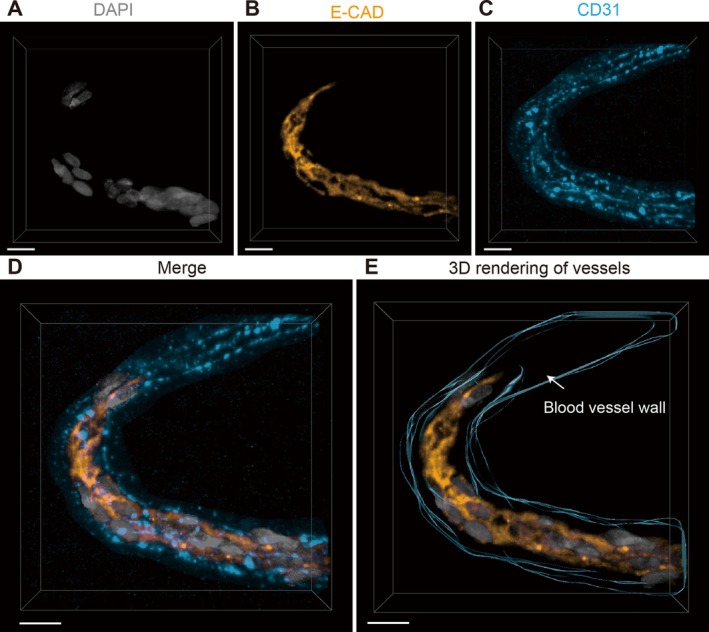
3D image of ectopic endometrial‐like structures found within blood vessels. (A) The nuclei of ectopic epithelial cells (DAPI). (B) Intra‐vascular ectopic epithelial cells (E‐cadherin). (C) Blood vessel (CD31). (D) Merged three‐channel visualisation. (E) 3D rendering of vessels. Scale bars: 30 μm (A–E). Ectopic epithelium (yellow), blood vessel (blue), nuclei (grey).

During 3D mapping of epithelial‐vascular interfaces, we unexpectedly identified that epithelial‐like cells existed within blood vessels in lesions in a limited number of OEM cases (*n* = 3). As depicted in Figures 7A–E and S5, a segment of the vascular lumen was occupied by epithelial‐like cell clusters. This finding represents a previously unreported morphological observation of epithelial‐like cell clusters within lesional vascular lumina.

### 2.6 Hormonal Status and Clinical Features Do Not Substantially Alter the Core Spatial Architecture of OEM Lesions

**FIGURE 8:**
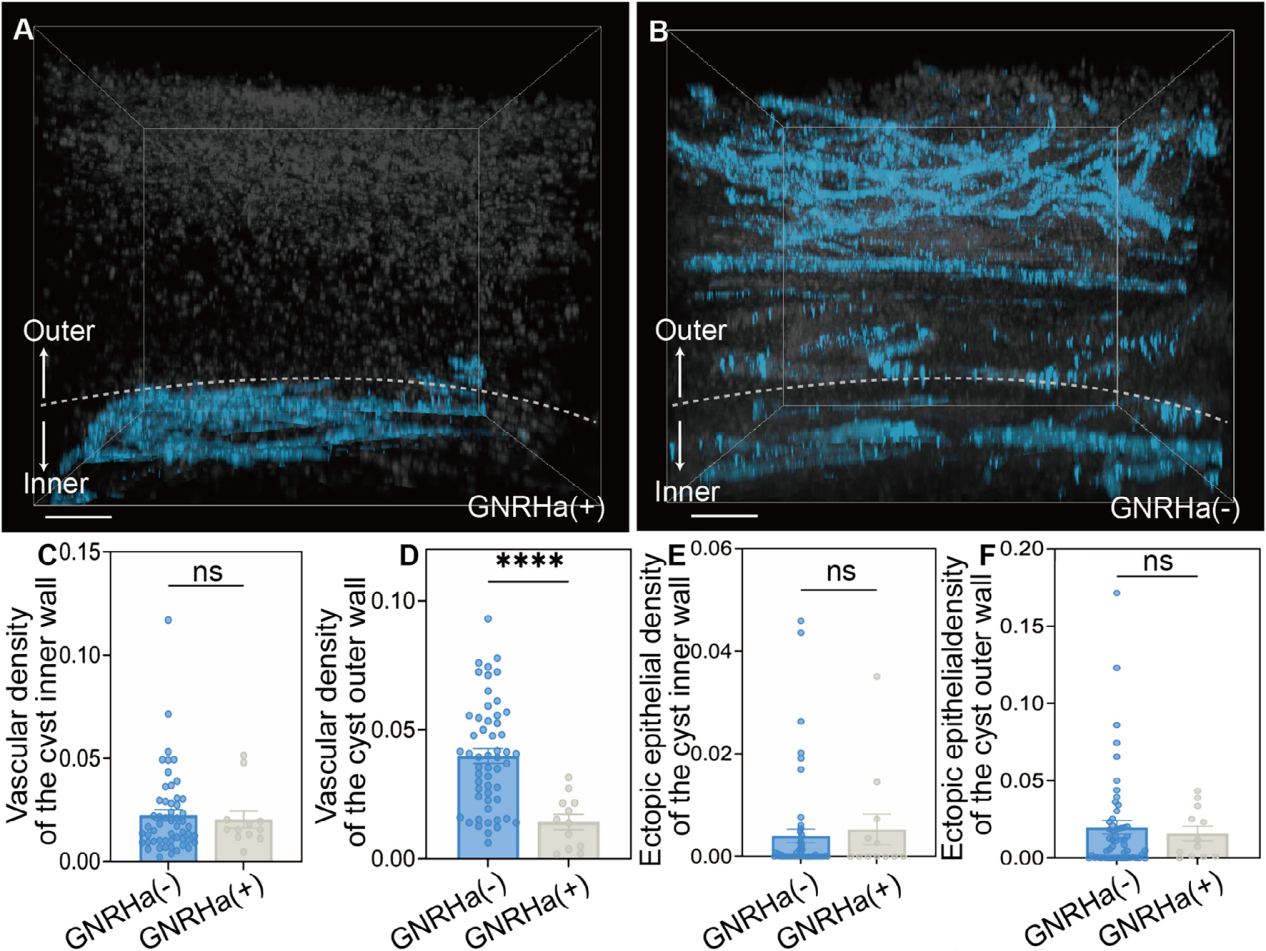
Characteristics of vascular density in the inner and outer walls of ovarian endometriotic cysts with and without GNRHa treatment. (A) 3D reconstruction of blood vessels in the inner and outer walls of ovarian endometriotic cysts in the GNRHa treatment group. (B) 3D reconstruction of blood vessels in the inner and outer walls of ovarian endometriotic cysts in the untreated group. (C) Comparison of vascular density in the inner walls of ovarian endometriotic cysts between the GNRHa treatment and untreated groups. (D) Comparison of vascular density in the outer walls of ovarian endometriotic cysts between the GNRHa treatment and untreated groups. (E) Comparison of ectopic epithelial density in the inner walls of ovarian endometriotic cysts between the GNRHa treatment and untreated groups. (F) Comparison of ectopic epithelial density in the outer walls of ovarian endometriotic cysts between the GNRHa treatment and untreated groups. (GNRHa(−), *n* = 27; GNRHa(+), *n* = 6, Scale bar: 100 μm). Data are presented as mean ± SEM; ***p < 0.001, ****p < 0.0001. The data were obtained from two randomly selected fields of view on the inner and outer cyst walls of each sample. Statistical analysis was performed using the Mann–Whitney *U* test. Blood vessel (blue), nuclei (grey).

To determine whether patient characteristics or hormonal status confounded the spatial organisation of ovarian endometrioma lesions, we analysed the associations between inner‐ and outer‐wall ectopic epithelial density, vascular density, and clinical variables, including age, cyst diameter, gravidity, parity, and number of pregnancies. No significant correlations were identified (Figures S6 and S7). Likewise, neither ectopic epithelial density nor vascular density differed significantly between proliferative‐ and secretory‐phase samples in either the inner or outer cyst wall. Preoperative gonadotropin‐releasing hormone agonist (GNRHa) treatment did not significantly alter ectopic epithelial density on either cyst wall (Figure 8E,F); however, vascular density on the outer cyst wall was significantly reduced in GNRHa‐treated patients compared with untreated controls (*p* < 0.0001), whereas vascular density on the inner wall remained unchanged (Figure 8A–D).

Beyond density measurements, we further assessed epithelial organisational diversity by quantifying, for each lesion, the number of distinct ectopic epithelial single‐cell morphotypes and 3D epithelial structural patterns. Neither cellular‐ nor structural‐level epithelial diversity showed significant associations with clinical variables or hormonal status, including menstrual cycle phase or GNRHa treatment (Figures S8 and S9). In addition, we consistently observed similar vascular architectural patterns across all clinical and hormonal subgroups examined. Specifically, vascular configurations consistently tracked with the spatial location of ectopic epithelium, with superficially located epithelium predominantly supported by unilateral vascular networks and deeply embedded epithelium more frequently associated with circumferential vascular arrangements, consistent with the location‐dependent vascular patterns described above.

## 3 Discussion

In this study, we employed an optimised tissue clearing method and 3D imaging to systematically map the spatial organisation of ectopic epithelium and vasculature in ovarian endometrioma lesions. This approach enabled visualisation of lesion architecture across multiple scales, from whole‐tissue organisation to single‐cell morphology, revealing structural features that are not readily appreciable using conventional two‐dimensional histology.

First, a key spatial feature identified in this study is the preferential enrichment of both ectopic epithelium and vasculature on the outer cyst wall compared with the inner wall (Figure 2B–D). This asymmetric distribution was consistently observed across samples and remained stable across patient age, cyst size, reproductive history, menstrual cycle phase, and preoperative hormonal treatment (Figures S6 and S7). These findings suggest that the outer cyst wall represents a spatially defined epithelial–vascular niche that is intrinsic to ovarian endometrioma architecture, rather than a secondary consequence of transient hormonal or clinical variables. From a clinical perspective, this spatial bias provides a structural framework for interpreting previous observations regarding lesion persistence and recurrence. For example, the predominance of ectopic epithelium on the outer wall may help explain why atypical epithelial features [32, 33] are more readily detected in certain sampling locations and why therapies targeting only the cyst lumen or inner wall, such as ethanol sclerotherapy [34, 35, 36] are associated with relatively high recurrence rates. Whilst our data do not directly test clinical outcomes, they highlight the importance of considering lesion spatial heterogeneity when evaluating diagnostic and therapeutic strategies [35].

Second, our study revealed an 87.8% detection rate for ectopic epithelium (Figure 3A), which is significantly higher than the 60.0% detection rate reported using traditional tissue sectioning techniques [29, 30]. However, it's important to note that this rate, based on analysis of only partial lesions from each patient, likely represents an underestimation of the true occurrence. Specifically, the limited extent of our analysis means that ectopic epithelium may exist in unexamined regions of the lesions, and that some lesions containing ectopic epithelium may have been missed entirely. Consequently, we postulate that the true detection of ectopic epithelium in ovarian endometriotic lesions is higher than our reported 87.8%, suggesting it is a common phenomenon.

Third, this ectopic epithelium displayed diverse morphological characteristics at both the tissue structure and cellular levels(Figures 3E–K and 4A1–F3), which closely parallels the genomic heterogeneity recently documented in ectopic epithelium [37]. Importantly, this morphological diversity was accompanied by distinct differences in epithelial proliferative activity. Ki‐67 analyses indicated that honeycomb, columnar, and irregular epithelial cells exhibited higher Ki67 positivity, whereas spot‐like, spherical, and spindle‐like morphotypes were consistently associated with low proliferative activity (Figure 4L). This association supports the interpretation that the observed heterogeneity reflects differences in functional epithelial states rather than random architectural variation or imaging perspective, without implying fixed lineage relationships or a hierarchical organisation amongst epithelial subpopulations. Notably, we observed multinucleation (such as spindle‐like and irregular shapes) in certain ectopic epithelial cells, suggesting aberrant proliferation and features that have previously been associated with aberrant proliferative behaviour in endometriosis‐related context [10, 38, 39, 40, 41]. Moreover, we identified spindle‐like ectopic epithelial cells characterised by significantly reduced E‐cadherin (E‐CAD) expression, a hallmark of epithelial‐mesenchymal transition (EMT) [10, 42, 43]. Whilst this observation is consistent with EMT‐like features, we do not infer invasive or malignant behaviour from morphology alone. Rather, these findings highlight the potential plasticity of ectopic epithelial cells and underscore the need for future studies incorporating transcriptomic and functional analyses, such as single‐cell sequencing, to clarify the biological significance of these phenotypes.

Fourth, we found that a substantial proportion of blood vessels in ovarian endometriotic lesions tend to grow around ectopic epithelium, with significantly higher vascular density surrounding ectopic epithelium compared to the stromal region (Figure 5). This pattern closely resembles the ‘epithelium‐vessel co‐localisation’ previously observed in murine endometrium [15], where blood vessels preferentially surround the endometrial epithelium, possibly due to the secretory function of the epithelium. In the normal endometrium, epithelial cells secrete various bioactive substances, essential for uterine function [44, 45], and these activities are supported by a dense vascular network [46, 47]. Similarly, ectopic epithelial cells secrete multiple bioactive substances, such as prostaglandins and cytokines, which are strongly implicated in pain, inflammation, and adhesion formation [48, 49, 50, 51]. These observations support the hypothesis that ectopic epithelial secretory activity may also depend on an enriched vascular microenvironment. Furthermore, we revealed distinct vascular configurations depending on the location of ectopic epithelium. Superficial ectopic epithelium was typically underlain by a unilateral vascular network, whilst deeply located epithelium was primarily encircled by vessels, forming a circumferential structure (Figure 6A–G). This spatially adaptive vascular organisation may increase epithelial–vascular interactions and facilitate metabolic and signalling exchange, thereby contributing to the persistence.

Unexpectedly, we observed endometrial epithelium‐like structures within the vascular lumina of endometrioma lesions (Figures 7A–E and S5), potentially providing novel morphological evidence to elucidate the mechanisms of disease metastasis. Whilst the exact pathogenesis of endometriosis remains incompletely understood, with several theories proposed (retrograde menstruation, coelomic metaplasia, and endometrial stem cell theory) [51, 52, 53, 54, 55, 56, 57], these do not fully account for the complex clinical manifestations. Previous researchers have suggested that vascular metastasis may contribute to the development of endometriosis [58, 59, 60]. Here, our observation represents a previously unreported morphological finding in human ovarian endometriosis (Figures 7A–E and S5). However, this finding remains descriptive in nature and does not establish active intravasation or hematogenous dissemination. Accordingly, it should be interpreted as a conceptual observation that warrants validation in larger cohorts and further mechanistic investigation.

Importantly, analyses across clinical variables and hormonal conditions indicate that the core epithelial–vascular spatial architecture described here is remarkably stable. Neither menstrual cycle phase nor preoperative GNRHa treatment substantially altered epithelial distribution or morphological diversity (Figures 8E,F, S7E,J, and 9E,F), although GNRHa treatment was associated with a selective reduction in vascular density on the outer cyst wall (Figure 8A–D), consistent with the known sensitivity of vascular compartments to hormonal deprivation [61, 62]. This dissociation suggests that epithelial organisation represents a relatively stable structural feature of ovarian endometriomas, rather than a transient reflection of hormonal status.

In summary, this study defines a stable, multi‐scale epithelial–vascular spatial architecture in ovarian endometriomas using three‐dimensional tissue clearing and imaging. These spatial, cellular, and vascular observations support a conceptual framework that integrates multi‐scale epithelial–vascular organisation in ovarian endometriomas (Figure S10). However, our study has certain limitations. First, due to the acquisition of only partial clinical samples, we were unable to achieve 3D imaging of the entire ovarian endometriotic cyst. Future imaging and 3D reconstruction of whole‐cyst tissues will be critical to more comprehensively elucidate the underlying mechanisms of pathogenesis. Second, the observation of endometrial epithelium‐like structures within the vascular lumina of endometrioma lesions was identified in three OEM cases. Although still infrequent, the recurrence of this finding across multiple cases supports that it is unlikely to represent an isolated imaging artefact, whilst remaining insufficient to establish a definitive mechanism. This significant finding requires validation in larger cohorts to determine its prevalence in OEM. Furthermore, mechanistic experiments are also needed to determine whether ectopic epithelial cells can indeed be transported via the bloodstream and subsequently implant in the distant sites. Third, although our tissue‐clearing and imaging workflow enables detailed spatial analysis, it remains time‐intensive and presents challenges for direct clinical translation [63, 64, 65]. The process often requires several weeks to complete, primarily due to limited antibody penetration in dense human tissues, as well as the extended demands of deep imaging and data reconstruction [18, 63, 66]. Finally, whilst this study focused on ectopic epithelium and vasculature, future work incorporating stromal and inflammatory components will be essential for a more comprehensive understanding of the lesion microenvironment and disease progression.

## 4 Conclusions

These findings collectively advance our understanding of ovarian endometriosis histopathology significantly, offering insights for clinical diagnosis and treatment.

## 5 Materials and Methods

### 5.1 Human Sample Collection

This study was approved by the institutional ethics review board of Medical Ethics Committee of Beijing Chaoyang Hospital Affiliated to Capital Medical University(2025‐department‐24). We recruited study participants at Beijing Chaoyang Hospital Affiliated to Capital Medical University in 2025, and all participants have signed informed consent forms. Inclusion criteria were: All patients were diagnosed as ovarian endometriosis by surgically and histologically, aged 24–53 years, and able to provide informed consent. Exclusion criteria were: (1) patients with acute infections, or suspicion of malignancy located in genital or extragenital area. (2) patients in whom clinical and paraclinical data were incomplete. (3) Patients were postmenopausal. We collected lesion samples from 49 patients meeting these criteria. Of these patients, 8 received preoperative GNRHa treatment for indications including fertility desire, anaemia, disease recurrence, pelvic pain, and reduction of intraoperative bleeding. All patients had a mean age of 37 years (range, 24–53 years) and cyst size ranging from 3 to 10.4 cm. Detailed patient information is provided in Table S2. Two normal endometrial tissues were obtained from women without endometriosis.

### 5.2 AI Technology Disclosure

All content within the submission is original work developed by the authors, without reliance on artificial intelligence‐generated material.

### 5.3 Tissue Clearing Procedure

#### 5.3.1 Modified CUBIC Method

Blocks of human ovarian endometriosis tissues were stored in formalin until use. First, the tissues were washed with phosphate‐buffered saline (PBS) for 6 h before clearing. Second, the tissues were immersed in CUBIC‐L, a mixture of 10 wt% *N*‐butyldiethanolamine (TCL, B0725) and 10 wt% Triton X‐100 (Sigma, X‐100), under shaking at 45°C for 3–4 days (depending on sample size). The CUBIC‐L solution was refreshed once during the delipidation process. Third, after 6 h of washing with PBS, the tissues were placed into 0.5 mL of immunostaining buffer, which was a mixture of PBS, 0.5% Triton X‐100, and 0.25% casein (Thermo Scientific, 37,582), containing 1:100 diluted anti‐CD31 antibody (Abcam, AB28364), E‐Cadherin antibody (NOVUS, AF748), and Ki‐67 (Thermo Scientific, 14‐5699‐82). This incubation lasted for 4–5 days (depending on sample size) at room temperature under gentle shaking. Fourth, the tissues were washed with PBS three times for 6 h. Fifth, the tissues were placed into 0.5 mL of immunostaining buffer (mixture of PBS, 0.5% Triton X‐100, 0.25% casein containing 1:100 diluted DAPI (Sigma, D9542), donkey anti‐rabbit IgG AlexaFluor647 (Thermo Scientific, A32795), donkey anti‐goat IgG AlexaFluor594 (Thermo Scientific, A11058), and donkey anti‐mouse IgG AlexaFluor488 (Abcam, 150,105)) for 3–4 days (depending on sample size) at room temperature under gentle shaking in the dark. Sixth, the tissues were again washed with PBS three times for 6 h. Finally, the tissues were transferred into a RIMS solution (20 g Histodenz (Sigma, D2158) + 15 mL PBS) and placed in the dark until tissues became completely transparent.

CUBIC‐R: Mixture of 45 wt% 2,3‐dimethyl‐1‐phenyl‐5‐pyrazolone (SAITONC, A70298), 30 wt% nicotinamide (SAITONC, N0078), and 5 wt% *N*‐butyldiethanolamine.

### 5.4 Compare the Crystallisation State of the Sample in CUBIC‐R and RIMS Reagents

To compare the crystallisation status of CUBIC‐R and RIMS reagents at different time points, we conducted experiments at 0 h, 0.5 h, and 2 h, respectively. And we captured overall image, and then captured detailed crystal images under Nikon Ci microscope.

### 5.5 Experimental Protocol for Haematoxylin–Eosin (HE) Staining of Paraffin‐Embedded Tissue Sections

Tissue blocks were cut into 5 mm × 5 mm × 2 mm pieces, fixed in 4% formaldehyde, dehydrated in a graded ethanol series, cleared in xylene, infiltrated with molten paraffin at 60°C, embedded, and sectioned. Paraffin sections were deparaffinised, rehydrated, stained with haematoxylin for 1 min, rinsed in running tap water for blueing, counterstained with eosin for 60 s, dehydrated, cleared, and mounted with neutral mounting medium before air‐drying at room temperature.

### 5.6 Method for Measuring the Light Penetration Depth of Treated Tissues With Two Clearing Agents at Different Time Points

Refractive index matching was conducted on the samples with CUBIC‐R and RIMS reagents, respectively. An equal volume (500 μL) of each reagent was added to the respective samples, and the light penetration depth at a wavelength of 405 nm was determined at 0 h, 0.5 h, 1 h, 2 h, 4 h and 12 h post reagent addition. All measurements were performed under identical laser parameter settings using a confocal laser scanning microscope. For the quantification of light penetration depth, the initial imaging depth was recorded when the laser beam first contacted the sample and a distinct sample signal was detected. The imaging depth along the *z*‐axis was then progressively increased until the sample signal in the field of view was completely undetectable, and the terminal depth was documented accordingly. The light penetration depth at 405 nm was defined as the absolute difference between the two recorded depth values.

### 5.7 Measurement of Fluorescence Retention Rate in Tissue Cleared With Two Reagents at Different Time Points During Prolonged Imaging

Tissues were subjected to two distinct tissue clearing protocols, that is the conventional CUBIC method and the modified CUBIC method developed in this study, to obtain cleared tissue samples. For the measurement of fluorescence signal retention rate during prolonged imaging, all experiments were conducted under fixed laser parameter settings and within a fixed field of view to ensure consistent detection conditions. Confocal images of the cleared tissues were acquired at 0 h, 0.5 h, 1 h, and 2 h during the prolonged imaging process. The fluorescence retention rate at each time point was calculated with the fluorescence intensity of the samples at 0 h set as the reference. Specifically, the fluorescence intensity of the samples at 0.5 h, 1 h, and 2 h was normalised to that at 0 h, and the corresponding percentage values were defined as the fluorescence retention rates at the respective time points.

### 5.8 Quantification of Ki‐67‐Positive Cells

To quantify the expression of Ki‐67 in ectopic epithelial cells with different morphological phenotypes, a standardised counting protocol was implemented. For each morphological subtype of ectopic epithelial cells, a minimum of 5 random fields were examined. At least 100 ectopic epithelial cells were counted per morphological subtype, and the number of Ki‐67 immunopositive cells was recorded. The Ki‐67 positivity rate for each morphological subtype was calculated as the ratio of Ki‐67‐positive cells to the total number of counted epithelial cells (≥ 100 cells per subtype). All quantifications were performed on images captured at 20× magnification under constant exposure and illumination settings.

### 5.9 Image Data Obtaining and Analysis

Clarified human ovarian endometriosis tissues were positioned upright on glass bottom culture dishes (NEST), and imaged using both a Zeiss Z1 LightSheet microscope and a Nikon C2 confocal microscope. Tile scans were stitched using ImarisStitcher (version 9.9.0, Bitplane) and Nikon software, and the imaging data were analysed with Imaris software (version 9.0.1, Bitplane). Then the concrete analysis was as follows: the CD31 signal and E‐Cadherin signal were used to generate a surface of the ectopic epithelium and blood vessels separately for quantitative analysis; Under the 3D surface module, according to the signals from CD31, E‐Cadherin, and DAPI to determine vascular volume, ectopic epithelial volume, and cleared ectopic tissue volume; The measurement of ectopic epithelial cells and their nuclei were under the Slice module; The ectopic epithelium, blood vessels, and nuclei pseudocolored yellow, blue, and grey, respectively; The Snapshot and Animation functions were used to capture images and videos, respectively.

### 5.10 3D Volumetric Analysis of Vascular and Ectopic Epithelial Density in Ovarian Endometriotic Cysts

A total of 49 samples of ovarian endometriotic cysts were collected from patients in this study. Amongst these samples, 27 samples in which the inner and outer cyst walls could be clearly distinguished were included for inner–outer wall–based quantitative analyses. For the 27 samples, random visual fields were selected separately for the blood vessels and ectopic epithelium located in the outer and inner walls of the cysts. To ensure the reliability and representativeness of the data, at least two 3D visual fields were acquired for both the blood vessel density and ectopic epithelium density in each of the inner and outer cyst walls. All acquired 3D visual fields were analysed using the Surface module of Imaris software. Vascular volume was defined based on the CD31 signal and ectopic epithelial volume was determined by the E‐cadherin signal. Vascular density, defined as the ratio of vascular volume to the total cleared ectopic tissue volume within the selected visual field, and ectopic epithelial density, defined as the ratio of ectopic epithelial volume to the total cleared ectopic tissue volume within the selected visual field.

### 5.11 Data Statistics

Quantitative data were subjected to analysis by using the Mann–Whitney *U* test (2 groups), and the Kruskal‐Wallis *H* test (a nonparametric alternative to one‐way ANOVA) with a post hoc multiple comparisons test for comparisons amongst more than two independent groups. Spearman's rank correlation analysis was performed to assess the correlations between variables. The results were analysed with GraphPad Prism 9.0.0 and presented as mean ± SEM; statistical significance was set as *p* < 0.05.

## Author Contributions

**Fanyuan Sun:** methodology, investigation, formal analysis, writing – original draft preparation, writing – review and editing. **Shuzhen Wang:** methodology, investigation, writing – review and editing. **Jinyan Zhao:** investigation, methodology, formal analysis. **Xili Zhu:** investigation. **Jinhua Leng**, **Chongdong Liu**, **Jing Chen**, **Jinming Guo**, **Bangjing Jiang**, **Haihe Zhou**, **Zhuoying Li**, **Hua Li:** writing – review and editing. **Menghui Li:** conceptualization, methodology, supervision, funding acquisition, writing – original draft preparation. **Yongcun Qu:** conceptualization, methodology, supervision, writing – original draft preparation, funding acquisition.

## Funding

This work was supported by the Young Elite Scientists Sponsorship Programme by BAST, Emerging Interdisciplinary Platform for Medicine and Engineering in Sports, the National Key R&D Program of China (2022YFC2704000) and the National Natural Science Foundation of China (Grant No. 82501949).

## Ethics Statement

This study was approved by the institutional ethics review board of the Medical Ethics Committee of Beijing Chaoyang Hospital Affiliated to Capital Medical University (2025‐department‐24).

## Conflicts of Interest

The authors declare no conflicts of interest.

## Supporting information


**Table S1:** Various morphologies of ectopic epithelium at structural level and the single cell types that constitute them.
**Table S2:** Clinical characteristics of the subjects.
**Figure S1:** Comparison of crystallisation of samples treated with CUBIC‐R and RIMS reagents at different times. (A–D) The samples treated with CUBIC‐R reagent at 0 h, 0.5 h, 1 h, and 2 h. (E–H) The samples treated with RIMS reagent at 0 h, 0.5 h, 1 h, and 2 h (The arrow indicates enlarged detail without crystallisation. The triangle indicates to enlarged details with crystallisation.) Scale bar: 5 mm.
**Figure S2:** Fluorescent and corresponding HE staining images of ectopic epithelium. (A1–D2) Fluorescent images and the corresponding HE stained sections of ectopic epithelium. Scale bar: 100 μm.
**Figure S3:** The layered structure of the walls of ovarian endometriotic cysts macroscopically. (A) Schematic diagram of the inner and outer cyst wall of ovarian endometriosis lesion tissue. (B) The inner and outer cyst wall of real ovarian endometriosis lesion tissue. Scale bars, 5 mm.
**Figure S4:** Ectopic epithelium exhibits diverse morphologies at structural level. (A–F) Respectively contiguous, papillary, curvilinear, clefted, discontinuous, and tubular‐like ectopic endometrial epithelium. Scale bar: 100 μm.
**Figure S5:** 3D image of ectopic endometrial‐like structures found within blood vessels. (A) The nuclei of ectopic epithelial cells (DAPI); (B) Intra‐vascular ectopic epithelial cells (E‐cadherin); (C) Blood vessel (CD31); (D) Merged three‐channel visualisation; (E) 3D rendering of vessels; (F) The nuclei of ectopic epithelial cells (DAPI); (G) Intra‐vascular ectopic epithelial cells (E‐cadherin); (H) Blood vessel (CD31); (I) Merged three‐channel visualisation; (J) 3D rendering of vessels. Scale bars: 30 μm (A–J). Ectopic epithelium(yellow), blood vessel(blue), nuclei(grey).
**Figure S6:** Correlation between vascular density (inner/outer cyst walls) and clinical features, and comparison of vascular density in different menstrual hormonal phases. (A–D) Correlation analyses of vascular density in the inner wall of cysts with cyst size (A), age (B), gravidity (C), and parity (D), respectively; (E) Comparison of vascular density in the inner wall of cysts between the proliferative phase and the secretory phase; (F–I) Correlation analyses of vascular density in the outer wall of cysts with cyst size (F), age (G), gravidity (H), and parity (I), respectively; (J) Comparison of vascular density in the outer wall of cysts between the proliferative phase and the secretory phase. (The data were obtained from two randomly selected fields of view on the inner and outer cyst walls of each sample. Statistical analysis was performed using the Mann–Whitney *U* test for comparisons between groups, and Spearman's rank correlation analysis was used to assess correlations between variables.)
**Figure S7:** Correlation between ectopic epithelial density (inner/outer cyst walls) and clinical features, and comparison of ectopic epithelial density in different menstrual hormonal phases (A–D) Correlation analyses of ectopic epithelial density in the inner wall of cysts with cyst size (A), age (B), gravidity (C), and parity (D), respectively; (E) Comparison of ectopic epithelial density in the inner wall of cysts between the proliferative phase and the secretory phase; (F–I) Correlation analyses of ectopic epithelial density in the outer wall of cysts with cyst size (F), age (G), gravidity (H), and parity (I), respectively; (J) Comparison of ectopic epithelial density in the outer wall of cysts between the proliferative phase and the secretory phase. (The data were obtained from two randomly selected fields of view on the inner and outer cyst walls of each sample. Statistical analysis was performed using the Mann–Whitney *U* test for comparisons between groups, and Spearman's rank correlation analysis was used to assess correlations between variables.)
**Figure S8:** Correlation of the number of distinct 3D epithelial morphologies per lesion with clinical characteristics and comparisons across menstrual hormone phases and GNRHa treatment status. (A–D) Correlation analyses of the number of distinct 3D epithelial morphologies per lesion with cyst size (A), age (B), gravidity (C), and parity (D), respectively; (E) Comparisons of the number of distinct 3D epithelial morphologies per lesion between proliferative and secretory phases; (F) Comparisons of the number of distinct 3D epithelial morphologies per lesion between the GNRHa untreated and treated groups. (Statistical analysis was performed using the Mann–Whitney *U* test for comparisons between groups, and Spearman's rank correlation analysis was used to assess correlations between variables.)
**Figure S9:** Correlation of the single‐cell level number of distinct ectopic epithelial cell morphologies per lesion with clinical characteristics and comparisons across menstrual hormone phases and GNRHa treatment status. (A–D) Correlation analyses of the single‐cell level number of distinct ectopic epithelial cell morphologies per lesion with cyst size (A), age (B), gravidity (C), and parity (D), respectively; (E) Comparisons of the single‐cell level number of distinct ectopic epithelial cell morphologies per lesion between proliferative and secretory phases; (F) Comparisons of the single‐cell level number of distinct ectopic epithelial cell morphologies per lesion between the GNRHa untreated and treated groups. (Statistical analysis was performed using the Mann–Whitney *U* test for comparisons between groups, and Spearman's rank correlation analysis was used to assess correlations between variables).
**Figure S10:** (A) Main finding 1: Outer vs inner cyst wall. (B) Main finding 2: 3D epithelial heterogeneity & proliferation. (C) Main finding 3: Epithelial–vascular spatial coupling. (D) Main finding 4: Intravascular epithelial‐like clusters. (E) Future plan.

**Video S1:** Original 3D image of branched tubular ectopic endometrial epithelium(yellow).

**Video S2:** 3D rendering of ectopic endometrial epithelium(yellow) and its surrounding blood vessels(blue).

**Video S3:** 3D rendering of vessels(blue) and ectopic epithelium(yellow) showing vascular patterns around superficial ectopic epithelium in OEM lesions.

**Video S4:** 3D rendering of vessels(blue) and ectopic epithelium(yellow) showing vascular patterns around deep ectopic epithelium in OEM lesions.

## Data Availability

The data that support the findings of this study are available from the corresponding author upon reasonable request.
