# Supplementary material for: Three‐Dimensional Architecture of Ectopic Epithelium and Vasculature in Ovarian Endometriosis Revealed by Tissue‐Clearing Imaging
Source: Cell Prolif. 2026 Mar 12;59(8):e70197. doi: 10.1111/cpr.70197 (PMC13325998; doi:10.1111/cpr.70197)
Supplement: Supplementary file 1 [file CPR-59-e70197-s003.docx]

**Table S1. Various morphologies of ectopic epithelium at structural level and the single cell types that constitute them.**

| **Various morphologies of ectopic epithelium at structural level** | **Single cell types that constitute the morphologies of various ectopic epithelium** |
| --- | --- |
| Contiguous structures | Honeycomb, irregular, spherical, columnar-like, spindle-like shapes |
| Papillary structures | Columnar-like and honeycomb shapes |
| Curvilinear structures | Columnar-like shapes and honeycomb shapes |
| Clefted structures | Columnar-like and honeycomb shapes |
| Discontinuous structures | Spot-like and honeycomb shapes |
| Tubular-like structures | Columnar-like and honeycomb shapes |

**Table S2. Clinical characteristics of the subjects.**

| Subject number | Cyst size  (cm) | Menstrual cycle | Ages | Gravidity | Parity |
| --- | --- | --- | --- | --- | --- |
| 1 | 8.5 | proliferative phase | 29 | 0 | 0 |
| 2 | 4 | proliferative phase | 41 | 1 | 1 |
| 3 | 7.3 | proliferative phase | 29 | 0 | 0 |
| 4 | 5.7 | proliferative phase | 26 | 1 | 0 |
| 5 | 9 | proliferative phase | 31 | 1 | 0 |
| 6 | 6.8 | proliferative phase | 40 | 0 | 0 |
| 7 | 5 | proliferative phase | 34 | 3 | 1 |
| 8 | 5 | proliferative phase | 45 | 0 | 0 |
| 9 | 6 | proliferative phase | 39 | 1 | 1 |
| 10 | 6.5 | proliferative phase | 29 | 0 | 0 |
| 11 | 6 | proliferative phase | 41 | 2 | 1 |
| 12 | 10 | proliferative phase | 38 | 0 | 0 |
| 13 | 8 | proliferative phase | 39 | 1 | 1 |
| 14 | 6 | proliferative phase | 41 | 2 | 1 |
| 15 | 5.1 | proliferative phase | 39 | 0 | 0 |
| 16 | 6.3 | proliferative phase | 35 | 0 | 0 |
| 17 | 10.9 | proliferative phase | 37 | 1 | 0 |
| 18 | 4.7 | proliferative phase | 42 | 2 | 0 |
| 19 | 7.8 | proliferative phase | 41 | 3 | 1 |
| 20 | 8.9 | proliferative phase | 29 | 0 | 0 |
| 21 | 8 | proliferative phase | 42 | 4 | 2 |
| 22 | 3 | proliferative phase | 53 | 1 | 1 |
| 23 | 5.3 | proliferative phase | 33 | 0 | 0 |
| 24 | 7.6 | proliferative phase | 40 | 1 | 1 |
| 25 | 9.5 | proliferative phase | 42 | 1 | 1 |
| 26 | 8.5 | proliferative phase | 29 | 0 | 0 |
| 27 | 6 | proliferative phase | 36 | 1 | 1 |
| 28 | 6 | secretory phase | 25 | 0 | 0 |
| 29 | 6 | secretory phase | 28 | 0 | 0 |
| 30 | 10.4 | secretory phase | 48 | 3 | 1 |
| 31 | 6.1 | secretory phase | 43 | 2 | 1 |
| 32 | 5.1 | secretory phase | 35 | 1 | 1 |
| 33 | 6 | secretory phase | 41 | 3 | 0 |
| 34 | 6.3 | secretory phase | 43 | 0 | 0 |
| 35 | 9 | secretory phase | 27 | 0 | 0 |
| 36 | 6.9 | secretory phase | 24 | 0 | 0 |
| 37 | 7.2 | secretory phase | 51 | 2 | 1 |
| 38 | 8.4 | secretory phase | 34 | 1 | 0 |
| 39 | 3.4 | secretory phase | 36 | 2 | 2 |
| 40 | 10.4 | secretory phase | 48 | 3 | 1 |
| 41 | 9.3 | secretory phase | 33 | 1 | 0 |
| 42 | 4 | GnRH-agonist treatment | 39 | 4 | 2 |
| 43 | 5 | GnRH-agonist treatment | 37 | 3 | 2 |
| 44 | 5 | GnRH-agonist treatment | 35 | 2 | 1 |
| 45 | 5.2 | GnRH-agonist treatment | 32 | 0 | 0 |
| 46 | 8 | GnRH-agonist treatment | 43 | 0 | 0 |
| 47 | 8 | GnRH-agonist treatment | 43 | 0 | 0 |
| 48 | 8.7 | GnRH-agonist treatment | 49 | 1 | 0 |
| 49 | 4.7 | GnRH-agonist treatment | 34 | 1 | 1 |

**Figure S1. Comparison of crystallization of samples treated with CUBIC-R and RIMS reagents at different times.**

(A-**D**) The samples treated with CUBIC-R reagent at 0h, 0.5h, 1h, and 2h.

**(E-H)** The samples treated with RIMS reagent at 0h, 0.5h, 1h, and 2h (The arrow indicates enlarged detail without crystallization. The triangle indicates to enlarged details with crystallization.) Scale bar: 5mm.

**Figure S2.** Fluorescent and corresponding HE staining images of ectopic epithelium.

**(A_1_-D_2_)** Fluorescent images and the corresponding HE stained sections of ectopic epithelium.

Scale bar: 100 μm

**Figure S3.** **The layered structure of the walls of ovarian endometriotic cysts macroscopically.**

(A) Schematic diagram of the inner and outer cyst wall of ovarian endometriosis lesion tissue.
(B) The inner and outer cyst wall of real ovarian endometriosis lesion tissue. Scale bars, 5mm.

**Figure S4. Ectopic epithelium exhibits diverse morphologies at structural level.**

**(A-F)** Respectively contiguous, papillary, curvilinear, clefted, discontinuous, and tubular-like ectopic endometrial epithelium. **Scale bar: 100 μm**

**Figure S5. 3D image of ectopic endometrial-like structures found within blood vessels.**

**(A)** The nuclei of ectopic epithelial cells (DAPI);

**(B)** Intra-vascular ectopic epithelial cells (E-cadherin);

**(C)** Blood vessel (CD31);

**(D)** Merged three-channel visualization;

**(E)** 3D rendering of vessels;

**(F)** The nuclei of ectopic epithelial cells (DAPI);

**(G)** Intra-vascular ectopic epithelial cells (E-cadherin);

**(H)** Blood vessel (CD31);

**(I)** Merged three-channel visualization;

**(J)** 3D rendering of vessels. Scale bars: 30 µm (**A-J**). Ectopic epithelium(yellow), blood vessel(blue), nuclei(gray).

**Figure S6. Correlation between vascular density (inner/outer cyst walls) and clinical features, and comparison of vascular density in different menstrual hormonal phases**

**(A-D)** Correlation analyses of vascular density in the inner wall of cysts with cyst size **(A)**, age **(B)**, gravidity **(C)**, and parity **(D),** respectively;

**(E)** Comparison of vascular density in the inner wall of cysts between the proliferative phase and the secretory phase;

**(F-I)** Correlation analyses of vascular density in the outer wall of cysts with cyst size (F), age (G), gravidity (H), and parity (I), respectively;

**(J)** Comparison of vascular density in the outer wall of cysts between the proliferative phase and the secretory phase. (The data were obtained from two randomly selected fields of view on the inner and outer cyst walls of each sample. Statistical analysis was performed using the Mann-Whitney U test for comparisons between groups, and Spearman's rank correlation analysis was used to assess correlations between variables.)

**Figure S7. Correlation between ectopic epithelial density (inner/outer cyst walls) and clinical features, and comparison of ectopic epithelial density in different menstrual hormonal phases**

**(A-D)** Correlation analyses of ectopic epithelial density in the inner wall of cysts with cyst size **(A),** age **(B),** gravidity **(C),** and parity **(D)**, respectively;

**(E)** Comparison of ectopic epithelial density in the inner wall of cysts between the proliferative phase and the secretory phase;

**(F-I)** Correlation analyses of ectopic epithelial density in the outer wall of cysts with cyst size **(F),** age **(G),** gravidity **(H),** and parity **(I),** respectively;

**(J)** Comparison of ectopic epithelial density in the outer wall of cysts between the proliferative phase and the secretory phase. **(**The data were obtained from two randomly selected fields of view on the inner and outer cyst walls of each sample. Statistical analysis was performed using the Mann-Whitney U test for comparisons between groups, and Spearman's rank correlation analysis was used to assess correlations between variables.**)**

**Figure S8. Correlation of the number of distinct 3D epithelial morphologies per lesion with clinical characteristics and comparisons across menstrual hormone phases and GNRHa treatment status**

**(A-D)** Correlation analyses of the number of distinct 3D epithelial morphologies per lesion with cyst size **(A)**, age **(B)**, gravidity **(C)**, and parity **(D)**, respectively;
**(E)** Comparisons of the number of distinct 3D epithelial morphologies per lesion between proliferative and secretory phases;

**(F)** Comparisons of the number of distinct 3D epithelial morphologies per lesion between the GNRHa untreated and treated groups. (Statistical analysis was performed using the Mann-Whitney U test for comparisons between groups, and Spearman's rank correlation analysis was used to assess correlations between variables.)

**Figure S9. Correlation of the single-cell level number of distinct ectopic epithelial cell morphologies per lesion with clinical characteristics and comparisons across menstrual hormone phases and GNRHa treatment status**

**(A-D)** Correlation analyses of the single-cell level number of distinct ectopic epithelial cell morphologies per lesion with cyst size **(A)**, age **(B)**, gravidity **(C)**, and parity **(D)**, respectively;

**(E)** Comparisons of the single-cell level number of distinct ectopic epithelial cell morphologies per lesion between proliferative and secretory phases;

**(F)** Comparisons of the single-cell level number of distinct ectopic epithelial cell morphologies per lesion between the GNRHa untreated and treated groups. (Statistical analysis was performed using the Mann-Whitney U test for comparisons between groups, and Spearman's rank correlation analysis was used to assess correlations between variables.)

**Figure S10**.

**Video S1.** Original 3D image of branched tubular ectopic endometrial epithelium(yellow).

**Video S2.** 3D rendering of ectopic endometrial epithelium(yellow) and its surrounding blood vessels(blue).

**Video S3.** 3D rendering of vessels(blue) and ectopic epithelium(yellow) showing vascular patterns around superficial ectopic epithelium in OEM lesions.

**Video S4.** 3D rendering of vessels(blue) and ectopic epithelium(yellow) showing

vascular patterns around deep ectopic epithelium in OEM lesions.
